# Maximizing performance in child footballers: chronotype and time of day study

**DOI:** 10.3389/fphys.2025.1591728

**Published:** 2025-10-17

**Authors:** Ilknur Kaba, Baha Engin Çelikel, Oğuzhan Adanur, Coşkun Yılmaz, Süreyya Yonca Sezer, Çetin Tan, Tebessüm Ayyıldız Durhan, Uğur Özer, Tülay Ceylan

**Affiliations:** ^1^ Medical School, Hitit University, Çorum, Türkiye; ^2^ School of Physical Education and Sports, Firat University, Elazığ, Türkiye; ^3^ Ministry of Youth and Sport, Ankara, Türkiye; ^4^ Kelkit Aydın Doğan Vocational School, Gümüşhane University, Gümüşhane, Türkiye; ^5^ Faculty of Sport Sciences, Munzur University, Tunceli, Türkiye; ^6^ Faculty of Sports Sciences, Gazi University, Ankara, Türkiye; ^7^ Faculty of Sports Sciences, Mehmet Akif Ersoy University, Burdur, Türkiye; ^8^ Faculty of Sports Sciences, Ondokuz Mayıs University, Samsun, Türkiye

**Keywords:** physical fitness, children’s health, respiratory function, exercise interventions, circadian rhythm, chronotype

## Abstract

**Background:**

A limited number of studies have investigated the effects of circadian rhythm-based running exercise interventions on physical fitness in child athletes. This study aimed to investigate the effects of an eight-week morning and evening running program on lower extremity strength, agility and respiratory function in 10–12-year-old male footballers.

**Methods:**

Participants visited the laboratory three times, with one-day intervals before and after the training program. The assessments included maximal inspiratory pressure (MIP), maximal expiratory pressure (MEP), forced expiratory volume in one second (FEV1), forced vital capacity (FVC), and the FEV1/FVC ratio. Additionally, agility and functional performance tests (FPTs) were conducted for both the dominant and non-dominant legs.

**Results:**

The findings indicated that morning running was more effective than evening running in enhancing respiratory function. Morning running also showed superior results in lower extremity strength tests, particularly in the single-leg (SL) and triple-leg (THD) crossover hop for distance tests (CHDs) and the 6-m timed-hop test (6 m THT). Furthermore, the agility performance of the morning running group was significantly better than that of the evening and control groups.

**Conclusion:**

Consequently, morning running interventions had a positive impact on key physical fitness parameters, including respiratory muscle strength, respiratory function, agility, and lower extremity strength in child footballers.

## 1 Introduction

Numerous studies had examined the effects of homeostatic changes on athletic performance throughout the daily cycle. These investigations had consistently revealed that athletes showed significant differences in performance depending on the time of day they trained (Brown et al., 2008; Chtourou et al., 2012a; Facer-Childs and Brandstaetter, 2015; Rae et al., 2015; Souissi et al., 2019). Circadian rhythm, which regulates physiological processes over a 24-h cycle, had been shown to influence athletic performance. Previous research had suggested that evening training had more favorable effects on performance compared to morning training (Hill, 2014; Pullinger et al., 2014; Fernandes et al., 2014; Ammar et al., 2015a; Belkhir et al., 2019; American College of Sports Medicine, 2000).

Long-term evening training that combined strength and endurance had been associated with greater increases in muscle hypertrophy and mass compared to morning training (Küüsmaa et al., 2016). These findings had been consistently observed across various fitness domains, including cardiovascular endurance tests (such as swimming and cycling) and strength-based assessments (such as countermovement jumps and isometric muscle contractions) (West et al., 2014). The variability in athletic performance had been strongly linked to circadian rhythm mechanisms, emphasizing its importance in designing training programs (Brown et al., 2008; Facer-Childs and Brandstaetter, 2015; Rae et al., 2015; Pullinger et al., 2014; Guette et al., 2005; Lericollais et al., 2011).

Circadian rhythms had been defined as 24-h cyclical patterns of physiological functions such as lung function, and had become a significant focus in both basic and clinical research. The regulation of these rhythms had been reported to be governed by the circadian clock located in the suprachiasmatic nucleus of the hypothalamus (Ralph et al., 1990). This system had been described as being coordinated by the hypothalamic-pituitary axis, the autonomic nervous system, and clock proteins through regulatory feedback loops (Cardone et al., 2005; Medarov et al., 2008). Circadian rhythms had been noted as endogenous biological oscillations present in all living organisms (Henst et al., 2015). The suprachiasmatic nucleus had been recognized as the primary circadian clock that aligned these rhythms with external cues such as light–dark cycles, meal timing, and social interactions (Kusumoto et al., 2021). Circadian rhythms in human physiology and behavior had been emphasized for their impact not only on quality of life but also on success in competitive sports. Rhythmic body activities and performance in athletes had been reported to produce significant outcomes in high-level competitions (Pradhan et al., 2024). Moreover, this system had been shown to regulate essential physiological processes such as the sleep–wake cycle, activity fluctuations, and skeletal muscle synchronization (Tahara et al., 2017).

Many circadian rhythm-related factors, including body temperature, chronotype (morning or evening type), training time, and daily biochemical variability, had been shown to contribute to diurnal variations in athletic performance (Lericollais et al., 2011; Kim et al., 2015; Ammar et al., 2015b; Knaier et al., 2016). Lung function had also been reported to exhibit circadian rhythmicity (Medarov et al., 2008).

Efficient pulmonary function had played a critical role in oxygen regulation during running, directly influencing running economy and overall performance (Thompson, 2017). Regular running training had been shown to significantly influence peak performance, with morning sessions yielding the greatest benefits. However, afternoon and evening training had also been reported to enhance neuromuscular adaptation by increasing the amplitude of daily physiological fluctuations (Chtourou and Souissi, 2012; Ayala et al., 2021).

Integration of Advanced Biomedical Technologies with Traditional Methods: The combination of classical biochemical and ergophysiological methods with innovative approaches such as telomere analysis, genotyping/phenotyping, and metabolomics had been found effective in evaluating children’s athletic performance (Spanakis et al., 2024). Physical activity and athletic performance had been described as complex phenotypes influenced by both environmental and genetic factors (Ahmetov et al., 2024). Prioritizing morning training in professional marathon running had been reported to improve performance outcomes (Henst et al., 2015).

Despite extensive research on circadian rhythm-based training interventions, most studies had focused on adult and adolescent athletes. There had been limited research investigating the effects of morning and evening running on key physical fitness parameters such as lower extremity strength, respiratory function, respiratory muscle strength, and agility in young athletes.

Therefore, this study had aimed to investigate the effects of 8 weeks of morning and evening running on lower extremity strength and respiratory function in 10–12-year-old male football players. It is predicted that running training performed in the morning and evening hours on male football players aged 10–12 years with different chronotypes will have different effects on lower extremity muscle strength, respiratory functions and agility levels, and that training performed in a time period appropriate to the individual’s chronotype will lead to more significant improvements in physical performance parameters.

## 2 Materials and methods

### 2.1 Participants

In this study, a parallel two-group pre-test–post-test randomized controlled trial was conducted according to CONSORT guidelines (Moher et al., 2001). The study protocol was registered at ClinicalTrials.gov (ID: NCT06817486). All participants and their parents were given detailed information about before the study, and written informed consent was obtained in accordance with the ethical principles described in the Declaration of Helsinki. Help was received from a third-level athleticism coach for planning and implementing running exercises to be used in the project. This study was designed considering the physiological changes of children and the pre-adolescent or early adolescence period. The study was designed according to the rules of the Declaration of Helsinki (World Medical Association, 2013) and approved by the ethics committee for scientific research of Gümüşhane University (at its meeting on 21.02.24 and number 2024/2; decision number E-95674917-108.99-239802).

### 2.2 Experimental design

**FIGURE 1:**
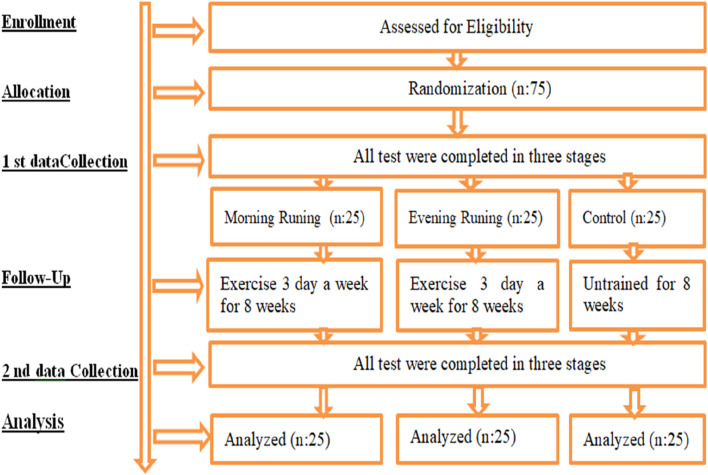
Experimental design.

The participants visited the laboratory on three separate occasions, with one-day intervals before and after the training period. During the initial visit, both the participants and their parents received comprehensive information about the study procedures, and pilot tests were administered. On the second visit, respiratory function tests were conducted, and measurements of respiratory muscle strength, height, and body weight were taken. During the third and final visit, the dominant leg was identified, and functional performance and agility tests were conducted for both the dominant and non-dominant legs. All tests were performed at the same time of day to minimize circadian variability. Participants were instructed to maintain a normal diet and sleep routine and to refrain from intense exercise for 24 h before testing. Additionally, they fasted for 3 hours prior to testing and consumed 500 mL of water 2 hours before the assessments (A.C.S.M. 2000). After completing the eight-week running training program, all measurements were repeated (Figure 1). To ensure consistency in sleep patterns throughout the study, participants were required to adhere to a structured sleep schedule, ranging from 9 to 12 h per night (Chaput et al., 2018).

### 2.3 Population and sample

The aim of this study was to investigate the effects of morning and evening running training on lower extremity strength, agility performance and respiratory function in 50 male football players aged 10-12 years who have been training regularly for at least 2 years. The control group consisted of 25 athletes. A total of 75 children aged 10-12 years participated in our study. Power analysis was performed with the G.Power 3.1 program to determine the sample size of the study and the d value was found to be 1.12 (α = 0.05, 1-β = 0.95, η2p = 0.8). As a result of the analysis, it was decided to include at least 25 participants in each group. The study was divided into three groups: morning running (MRI), evening running (ERG) and control (CON). Our study included individuals who had been doing sports for 2 years. No medication was used before the study. Individuals with illness or disability were excluded from the study.

### 2.4 Instruments

The participants’ height was measured using a standardised height gauge (Seca, Germany) with an accuracy of 0.1 cm, while leaning against a wall without shoes. Their body weights were recorded before and after the 12-week sports training programme using a digital scale (Beurer, model GS27) with an accuracy of 0.1 kg. The participants wore minimal clothing and no footwear during the weighing process.

#### 2.4.1 Chronotype

The morningness-eveningness scale for children (MESC) created by Carskadon et al. (1993), aims to determine the daily preferences of school-age children. Comprising 10 items with four or five response options, scores range from 10 to 43. 21 points and 35 points are the cut-off points of the scale (Carskadon et al., 1993). The higher the score on the scale, the more children prefer mornings. A score of 21 or below indicates an evening type, 22–34 points indicates an moderate type and 35 points and above indicate a morning type. The Turkish version’s validity and reliability were established by Önder and Beşoluk (2013).

#### 2.4.2 Lower extremity strength tests

**FIGURE 2:**
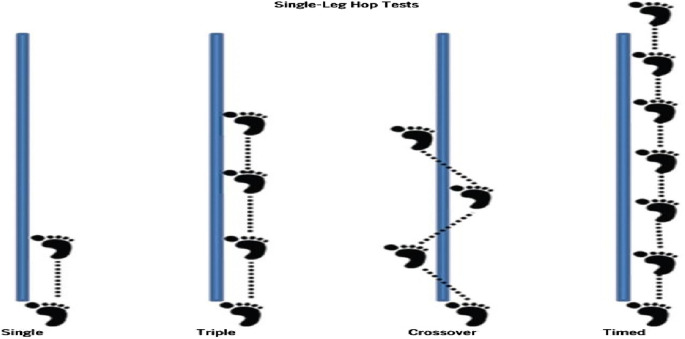
Application of functional performance tests (Schmitt et al., 2012).

Dominant and nondominant foot measurements were taken for functional performance tests (FPTs), which were used to determine the subjects’ lower extremity strength. Prior to each test, the subjects were instructed on how to perform the measurement. Three trials were performed for each test prior to the actual measurements. After the trial repetitions, the participant was subjected to 3 main tests, and the success criterion in the test was determined as the subject landing on one leg with full stabilization and staying there for 3 seconds. The subjects rested for 30 s between trials. Arm movement was allowed during the movement, and no restrictions were imposed (Munro and Herrington, 2011). For all the trials, a 30 cm strip was drawn on the ground as a starting point, a 6 m long and 15 cm wide strip was placed vertically on the ground from the center of this strip, and all the measurements were taken on this platform (Figure 2).

Single Leg Hop for Distance (SL): In the SL test, subjects start standing on one leg at the marked starting line and, when ready, jump horizontally and as far as they can jump so that they fall on the same leg; the result is determined by the successful attempt between the starting line and the subject’s heel and recorded in cm (Schmitt et al., 2012).

Triple Hop for distance (THD): In the THD test, the subject began by standing on one leg at the start line and, when ready, jumped horizontally as long as he could three times in succession without stopping. The distance between the starting line and the heel height of the subject’s fall was recorded in cm (Schmitt et al., 2012).

Single Leg 6 m. Timed Hop Test (6 mt THT): The subject stands on one foot at the start line and finishes the 6-m track in the fastest possible time. The test began at the start line and ended when the subject’s heel touched the first point at which the subject crossed the finish line. All the subjects were tested three times, with a rest period of 2 min between each test. The test was timed in seconds using a standard stopwatch. The best time from the three trials was recorded in seconds. The use of arm movements during movement was allowed, and no restrictions were imposed (Yılmaz and Kabadayı, 2022).

Crossover Hop for Distance (CHD): The subject stands on one foot at the starting line and performs 3 jumps forward, and the distance jumped is recorded in cm. The first jump starts diagonally opposite the foot used and continues laterally to the side of the fall. For each test, the subjects were given three repetitions. The criterion for success in the test was that the subject landed with full stabilization on the leg and remained standing for 3 seconds. The best jump distance was recorded in cm. The subjects were given a 30 s rest interval between each trial (Yılmaz and Kabadayı, 2022).

#### 2.4.3 505 agility test

This test consists of measuring the time taken to complete the last 5 m of a 15 m track. The time within the first 10 m from the start of the test is not included in the test score. When the next 5 m distance is passed for the first time, the recording begins and stops when the same distance is returned (Nimphius et al., 2016).

#### 2.4.4 Measurement of height and weight

A Seca 769 electronic height measuring device (Seca Anonim Şirketi, Hamburg, Germany) was used. The device measures height with an accuracy of 0.1 cm and body weight with an accuracy of 0.01 kg. Body weight was measured in kilograms (kg) without shoes and wearing shorts and a T-shirt to avoid influencing the participants’ weight. Height was measured in centimeters (cm) without shoes, with the body weight evenly distributed on both feet (Table 1).

**TABLE 1:** Characteristics of participants.

Descriptives	MRG (n:25)		ERG (n:25)		CON (n:25)	
	X	Sd	X	Sd	X	Sd
Age (year)	11.00	0.50	11.28	0.74	11.16	0.69
Height (cm)	146.80	6.49	147.40	7.80	147.16	9.27
Body weight (kg)	36.56	6.68	37.64	7.64	36.68	6.21

Morning running group (MRG), evening running group (ERG), control group (CON).

#### 2.4.5 Pulmonary function tests

FEV1, FEV1/FVC (Tiffenau index), and FVC capacity were analyzed via a CPFS/D USB spirometer from MGF Diagnostics (Saint Paul, Minnesota, United States). Measurements were taken between 15:00 and 17:00 for all participants to obtain the highest spirometric throughput (Medarov et al., 2008). Participants with FEV1/FVC <75%, any chronic or pulmonary disease, medication that could affect lung function, or a history of upper respiratory tract infection were excluded from the study. Lung function tests were performed with the participants in the standing position. During the tests, the participants wore a nose clip and were instructed to hold their lips tightly around the mouthpiece to prevent air from escaping.

#### 2.4.6 Respiratory muscle strength

Maximal inspiratory pressure (MIP) and maximal expiratory pressure (MEP) were measured via a hand-held portable oral pressure meter (MicroRPM, CareFusion Micro Medical, Kent, United Kingdom) according to the American Thoracic Society and European Respiratory Society guidelines (American Thoracic Society, 2002). With the appropriate filters and holders in place, the nasal airway was closed with a clip. The mouthpiece assembly included a 1 mm hole to prevent glottic closure and minimize the contribution of the buccinator muscles during inspiration. Inspiratory and expiratory maneuvers were performed in the standing position, with MIP and MEP measurements started at the residual volume and total lung capacity, respectively, and continued for at least 1 s. The measurements were repeated until there was a 5% difference between the 2 best results, and the results were recorded as the mean cm H2O (Polkey et al., 1995).

#### 2.4.7 Running training

The morning running sessions were conducted between 08:00 and 10:00, while the evening sessions took place between 18:00 and 20:00 (Bessot et al., 2014). The exercise intensity for each participant in the running group was set at 50% of their heart rate (HR), calculated using the Karvonen formula: Target HR = [(220−age−resting HR) × intensity]+resting HR. HR was monitored from the first week of training using a telemetric heart rate monitor (Polar M400, Finland). Since environmental conditions can influence airway epithelial responses during high-intensity exercise (Boukelia et al., 2017), all participants performed continuous running exercises on a football field in Kelkit, Gümüşhane, Turkey (altitude: 1373 m). Each session lasted 50 min, including a 10-min warm-up and cool-down, and was performed 3 days per week over an eight-week period at the designated target HR. All sessions were supervised by trained coaches. The warm-up and cool-down routines incorporated static stretching and light exercises targeting the relevant muscle groups. Coaches were responsible for monitoring the athletes’ running technique and pace, ensuring safety, and providing motivation. To prevent dehydration, both groups were given adequate water throughout the sessions (Maresh et al., 2006).

### 2.5 Statistical analysis

Statistical analyses were performed via SPSS (Version 27.0 for Windows, Chicago, IL, United States) software, with the statistical significance set at 0.05. The Kolmogorov‒Smirnov normality test was performed to determine the homogeneity of the sample. Repeated measures two-way analysis of variance and Bonferroni correction were used to analyze differences in 1RM and diaphragm thickness measurements between trials. Furthermore, the effect size in pairwise group comparisons was calculated using partial eta-squared (η p 2). The interpretation of the parameter η p 2 is as follows: small values, such as 0.01, indicate a small effect size; medium values, such as 0.06, indicate a medium-sized effect; and large values, such as 0.14, indicate a strong effect (Richardson, 2011).

## 3 Results

**FIGURE 3:**
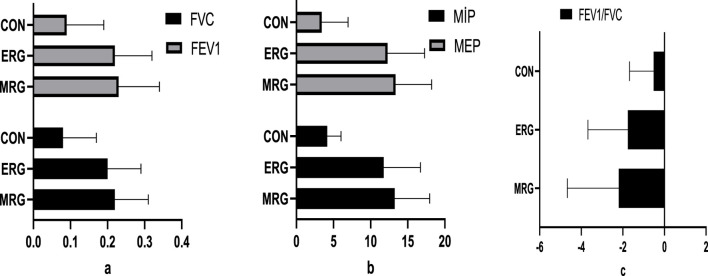
Comparison of respiratory function difference values.

The results of our study indicated that morning running was more effective than other types of exercise in improving respiratory function. Participants in the morning running group showed greater improvements in forced vital capacity (FVC) (10.18%), forced expiratory volume in one second (FEV_1_) (11.16%), maximal inspiratory pressure (MIP) (15.80%), and maximal expiratory pressure (MEP) (14.55%) compared to the other groups (p < 0.001; Figure 3).

**FIGURE 4:**
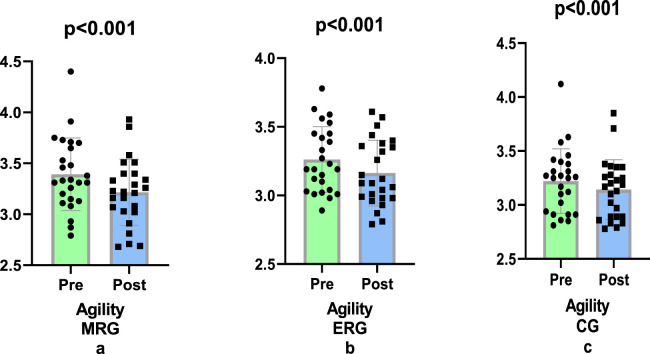
Comparison of agility difference values pre and post training.

In our study, it was found that agility scores improved more in the morning running group (−5.01%) compared to the evening (−3.06%) and control (−2.48%) groups following the 8-week running training program (p = 0.001, ηp^2^ = 0.186; Figure 4).

**FIGURE 5:**
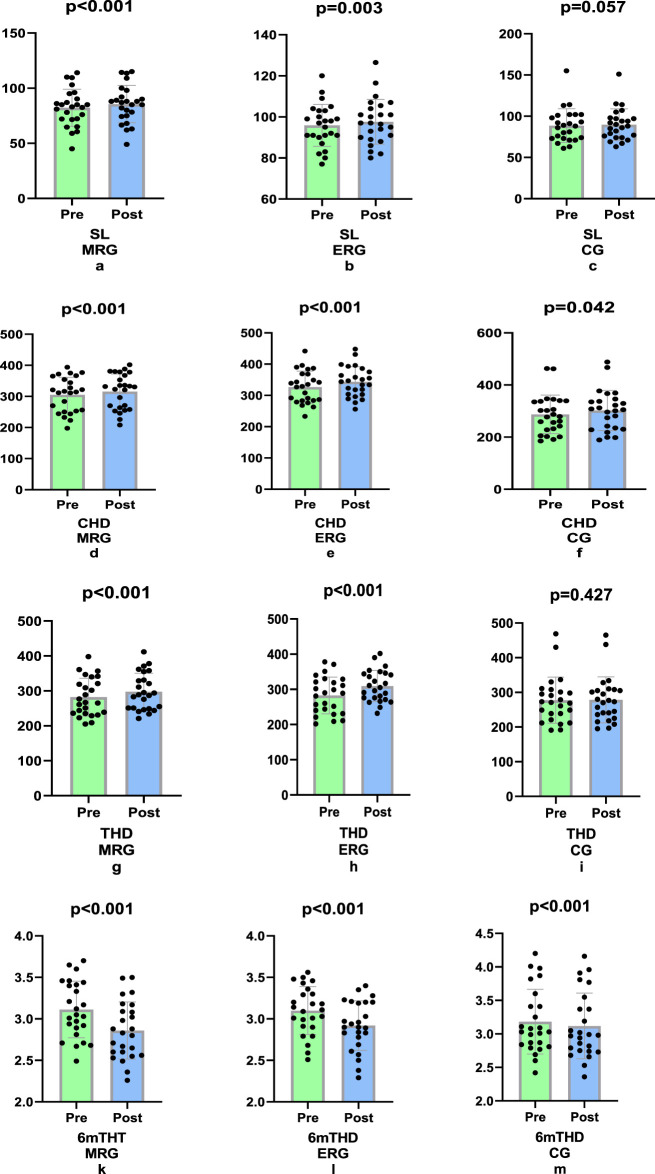
Comparison of difference values of lower extremity strength tests (non-dominant) a) Single leg (SL), triple leg (THD) and crossover hop for distance tests (CHD) and for distance tests 6 m timed-hop test (6 m THT).

In our study, morning running was more effective in the SL (4.07%, p < 0.001, ηp^2^ = 0.133) and 6 m THT (8.03%, p < 0.001, ηp^2^ = 0.639) tests, and evening running was more effective in the THD (5.16%, p < 0.001, ηp^2^ = 0.578) test; these differences were significant (p < 0.001). In the CHD test, no significant difference was found in any of the groups (p = 0.543, p < 0.001, ηp^2^ = 0.017; Figure 5).

**FIGURE 6:**
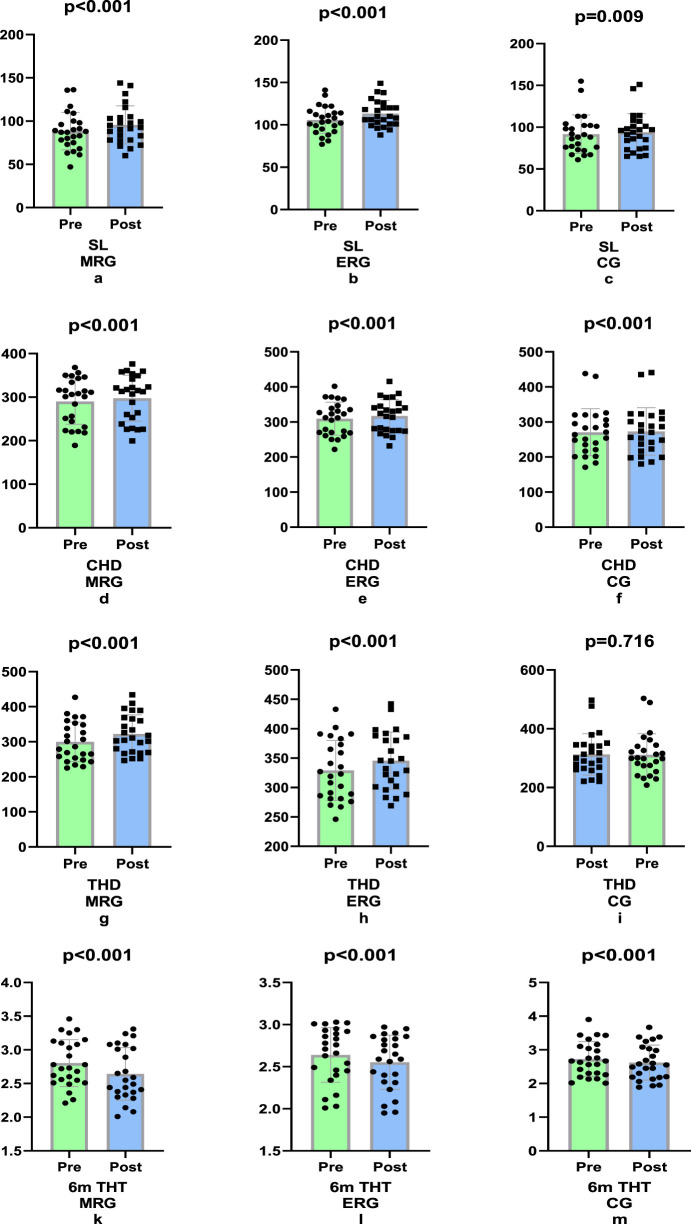
Comparison of the difference values of the lower extremity strength tests (dominant) Single leg (SL), Crossover (CHD), Triple leg (THD) and for distance tests 6 m timed-hop test (6 m THT).

In our study, it was found that morning jogging caused more improvement in the dominant leg in SL (8.15%, p < 0.001, ηp^2^ = 0.675), THT (2.49%, p < 0.001, ηp^2^ = 0.296), CHD (7.44%, p < 0.001, ηp^2^ = 0. 462) and 6 m THT (−5. 71%, p < 0.001, ηp^2^ = 0.236) caused more improvement in the dominant leg in lower extremity strength tests (p < 0.001; Figure 6).

## 4 Discussion

The ability of athletes to perform at their optimal level is a critical determinant of success in any sporting endeavor. To achieve peak performance, it is essential to identify the most effective training periods based on the body’s physiological and psychological capabilities (Atkinson and Reilly, 1996; Manfredini et al., 1998). A deeper understanding of the impact of circadian rhythms on performance, particularly in young athletes, can provide valuable insights for sports scientists and coaches, ultimately enhancing training efficiency and athletic outcomes. Accordingly, this study examined the effects of running training on lower extremity strength and respiratory function in children, taking diurnal variations into account. Since the sample of this study consisted only of boys who participated in regular soccer training, it is not possible to directly generalize the findings to athletes of different genders or to untrained children and elite young athletes. Physiological differences depending on gender and training level may alter adaptive responses to similar interventions. Therefore, it is important to test similar protocols with more heterogeneous samples in future studies to increase external validity.

The most significant findings of this study indicate that morning running had a more pronounced effect on respiratory parameters, agility, and lower extremity strength than evening running in male football players aged 10–12 years. Over the course of the eight-week training program, conducted in alignment with diurnal variations, the greatest improvements in respiratory function were observed in the morning running group. Specifically, forced vital capacity (FVC) increased by 10.18%, forced expiratory volume in one second (FEV_1_) by 11.16%, maximal inspiratory pressure (MIP) by 15.8%, and maximal expiratory pressure (MEP) by 14.55%.

Silva et al. (2006) conducted a study on asthmatic children and found no circadian rhythm-related changes in FEV_1_ values (Silva et al., 2006). However, the morning running group showed greater improvement in the 9-min run test compared to the control and evening groups. Boukelia et al. (2018) recommended that athletes train and compete in the morning, especially in hot and humid conditions, to minimize homeostatic disruptions. Despite this, their findings suggested no significant diurnal variations in lung function parameters such as FVC, PEF, FEV_1_, FEF 25%–75%, and the FEV_1_/FVC ratio (Boukelia et al., 2018). Another study examining the influence of circadian rhythm on anaerobic performance and recovery reported no significant differences in respiratory muscle strength or SaO_2_ levels across three different time points (Ünver and Atan, 2021). Similarly, Spengler and Shea (2000) observed minor but statistically significant circadian fluctuations in FEV_1_ and the FEV_1_/FVC ratio in healthy adults, whereas FVC and PEF remained unaffected by diurnal variations (Spengler and Shea, 2000).

Boukelia et al. (2017) reported no statistically significant differences in lung function tests conducted on professional athletes running between 9:00 a.m. and 2:00 p.m. However, morning trials performed under cold conditions induced greater physiological strain compared to evening trials, likely due to additional stressors such as fasting and exercise. Despite this, such stress was identified as a beneficial strategy for improving running performance and maintaining long-term health (Boukelia et al., 2017). Additionally, Gaultier et al. (1977) identified a circadian rhythm in lung resistance (R_1_) and dynamic lung compliance (C_1_dyn) in two groups of healthy children, with peak measurements recorded in the morning at 07:30, 11:30, 16:30, and 22:30 (Gaultier et al., 1977).

The findings of the present study further suggest that the diurnal fluctuations in respiratory parameters and lower limb strength observed in the morning running group persisted beyond the completion of the training program. These results indicate that intraoral temperature variations alone do not fully explain the time-of-day effects on anaerobic performance. This aligns with the findings of Martin et al. (2015), who demonstrated that diurnal variations in muscle strength persisted despite artificially warming the adductor pollicis muscle by 5°C in the morning (Martin et al., 2015).

In th literature, it has been suggested that reduced cortisol levels in the morning may limit athletic performance and delay peak performance attainment (Facer-Childs and Brandstaetter, 2015). While these findings imply that a certain period is required to reach optimal temperature and hormonal conditions for maximal athletic performance, cortisol may also play a supportive role in enhancing performance (Fernandes et al., 2014; Sedliak et al., 2007). This discrepancy in the literature highlights the importance of considering multiple factors, including chronotype, training regimen, experience, and the physiological state of the athlete, when designing training programs (Kusumoto et al., 2021; Roveda et al., 2020; Vidueira et al., 2023)

The agility scores of the participants who engaged in our study for a period of 8 weeks, during which they underwent running training, revealed that the group who undertook their running in the morning exhibited superior agility performance to both the evening group (1.95%) and the control group (2.53%). This difference was statistically significant (p = 0.001).

Huguet et al. (1995) reported that morning (8:30–10:30) runs were more effective in 9–11-year-old children in standing and seated sprint runs, according to the other time of day. Furthermore, they reported that the peak time performance of children would differ from that of adults (Huguet et al., 1995). In their 2012 study, Souissi and colleagues investigated the impact of time-specific training on daily performance fluctuations in boys (10-11 age). They reported that training programs conducted in the morning (7:00–8:00) and evening (17:00–18:00) groups demonstrated increased muscle strength and power gains following training, particularly during the morning hours. Furthermore, the anaerobic performance of the male subjects was superior to that of the female subjects in the morning training programs (Souissi et al., 2012).

The time-of-day differences were reported to vary between 3.4% and 10.2% across the studies, with the magnitude of the difference largely dependent on factors such as the measured performance variable, exercise mode, sprint duration, recovery type, number of sprint repetitions, and training status of the subjects (Pullinger et al., 2020).

The data indicate that short-term performances (i.e., those occurring in less than 1 minute) exhibit a consistent pattern: they are better in the afternoon (16:00–20:00) than in the morning (06:00–10:00). This hypothesis posits that intraoral temperature is a predictor of diurnal fluctuations and that the effects of time of day on short-term exercise performance can be mitigated by factors such as short-term exposure to moderately hot and humid environments; active warm-up protocols; intermittent fasting conditions; warming while listening to music; and prolonged training at a particular time of day. However, the hypothesis also fails to consider the impact of environmental conditions and the training programme on performance, as well as the grouping of training groups according to chronotype. This is because short-term maximal exercise performance throughout the day is regulated not only by body temperature, hormone levels, motivation and mood but also by a complex circadian system within skeletal muscle (Mirizio et al., 2020). Furthermore, the impact of elevation on the results is unclear. However, there is evidence that anaerobic strength is influenced by elevation (Banister and Woo, 1978).

It seems reasonable to posit that the time-of-day-specific effect may be explained by hormonal and neuromuscular adaptations to running training. A notable reduction in serum cortisol levels has been documented following morning training sessions (Sedliak et al., 2007; Kanaley et al., 2001). This evidence corroborates the notion that cortisol exerts short-term effects on the neuromuscular system, thereby influencing human performance. Moreover, the most well-known effect of cortisol is its role in skeletal muscle protein remodeling, particularly affecting type II muscle fibers (Crewther et al., 2011). Increased concentrations of the hormone cortisol in the morning may have a beneficial effect on an athlete’s overall performance during a competition (Deneen et al., 2017). However, the authors posit that there should not be disparate neural or mechanical mechanisms throughout the day but rather diurnal variations in endocrine secretions. A greater release of anabolic and catabolic hormones at a specific time of day (morning and evening) may result in enhanced outcomes. Thus far, no studies have been conducted on children that support this assertion. A number of potential mechanisms may be involved, but a detailed discussion would be based on speculation, as the mechanisms are beyond the scope of this experiment. Consequently, further research, which may encompass hormonal assessments, is imperative to ascertain the mechanisms through which running training may enhance anaerobic performance in children following evening or morning training sessions (Souissi et al., 2012).

The results of our study indicate that the SL (8.15%), THT (7.44%), CHD (2.49%) and 6 mt THT (5.71%) morning runs were more effective in lower extremity power tests than the other groups were, with significant differences observed between them. Considering the non-dominant leg, it was determined that morning running was more effective in the SL (4.07%) and 6 mt THT (8.03%) tests, whereas evening running was more effective in the THD (5.16%) test. There were significant differences between the groups. In the CHD test, although the highest rate of change was observed in the evening running group (9.50%), no significant difference was identified across all groups.

Souissi et al. (2012) reported that following a six-week pediatric resistance training programme, there was a greater increase in muscle strength and power in the morning hours than in the evening hours. Furthermore, there was a greater increase in muscle strength and power in morning training than in training specific to a certain time of day (Souissi et al., 2012). Some studies yielded different results than our study. For example, Guette et al. (2005) reported that the peak power (torque) value of both legs at 18:00 in the dominant and nondominant quadriceps femoris maximum voluntary contractions at different times of the day was statistically significantly highest (Guette et al., 2005). Küüsmaa et al. (2016) also examined the maximal voluntary contraction levels of the morning and evening groups in their study and demonstrated that there was a significant difference in favor of the evening group (Küüsmaa et al., 2016). Chtourou et al. (2012b) reported a difference in favor of the evening group in their study, which was programmed as a 8-week lower extremity strength training program and a 2-week taper training program (Chtourou et al., 2012b).

In contrast to the aforementioned studies, Chtourou et al. (2012c) investigated the daily strength differences between morning and evening groups following an 8-week lower extremity strength training programme. Their findings indicated that there were significant increases in strength from the morning to the evening before the commencement of the training programme. However, the daily differences in anaerobic power were blunted after the training programme, and no significant difference was observed between the morning and evening (Chtourou et al., 2012c).

Sedliak et al. (2007) reported that healthy male subjects exhibited diminished diurnal fluctuations in the isometric strength of the knee extensors following a 10-week morning training regimen. While there was a decrease in morning cortisol levels in the experimental group, there was no concomitant change in testosterone levels. These findings indicate that cortisol, but not testosterone, is the primary regulatory hormone of athletic performance and that reduced morning cortisol levels are associated with enhanced performance (Sedliak et al., 2007). Roveda et al. (2020) reported superior performance for M-types in the morning and E-types in the evening when football-specific motor skills were examined (Roveda et al., 2020). Nèji et al. (2021) reported that leg strength, speed and postural control are affected by time of day. The results demonstrated that leg strength, speed and postural control were significantly better in the afternoon than in the morning (Nèji et al., 2021).

In a study conducted by Di Cagno et al. (2013), no significant correlation was detected between the time of day and the performance of both elite and sedentary youth in motor coordination and reactive power tests. However, it is plausible that the time of day at which athletes typically engage in training for these skills may exert an influence. In practice, he recommended that coaches organize sport selection on the basis of reactive power and utilize the morning hours for untrained adolescents when the benefits of wakefulness and sleep can enhance performance (Di Cagno et al., 2013).

The mean body temperature decreases with age. It has been proposed that there are age-related differences in body temperature, with older individuals exhibiting lower temperatures and adolescent individuals exhibiting higher temperatures. This phenomenon can be attributed to the state of thermoregulation (Waalen and Buxbaum, 2011). Prior research has corroborated the correlation between body temperature and strength (Racinais et al., 2005; Racinais et al., 2004). The majority of articles that posit a correlation between daily temperature increase and enhanced performance, citing this as a passive warm-up, have been conducted with adult participants (Kusumoto et al., 2021; Racinais, 2010). However, it has been established that there is less variation in body temperature in children than in adults (Ahmed et al., 2008). Furthermore, children utilize disparate lower extremity joint functions than adults do (Beerse and Wu, 2022). These findings provide a potential explanation for the discrepancy between our study results and those reported in the literature. A further distinction pertains to the FPT tests, which represent the measurement protocol for lower extremity strength in the present study. In these tests, it is widely accepted that the standing long jump is an excellent functional measure of explosive lower extremity strength, which is significantly associated with health in children and adolescents (Tomkinson et al., 2021). The FPTs employed in the present study revealed that muscle activations differ in isokinetic knee strength tests, which is the measurement protocol of lower extremity strength most commonly utilized in the literature (Yılmaz and Kabadayı, 2022).

It is hypothesized that undertaking training in the morning has a similar effect to resistance training and enhances leg strength in athletes with an M-type chronotype. In this instance, the application of a high force to the ground with the dominant leg results in an increase in the horizontal force value, which subsequently leads to an increase in the stride length in the forward direction with the dominant leg. The application of low force to the ground with the nondominant leg has been shown to diminish the positive effect on stride length. Consequently, the impact of nondominant leg force on FPT tests was found to be inconsistent (Kusumoto et al., 2021).

This study has several limitations that should be considered. Firstly, it was assumed that all participants achieved maximal performance during the tests. However, due to the non-homogeneous distribution of player positions, this variable was not taken into account during the application and analysis phases. The participant group consisted solely of male child football players aged 10–12 years who had been engaged in regular training for at least 2 years, which limits the generalizability of the findings to broader age ranges and both genders. Moreover, the sample size was limited due to the specific characteristics of the study population; future studies with larger sample sizes may provide more reliable and generalizable results. Although the eight-week intervention period was sufficient to evaluate short-term effects, longer-term follow-up studies are needed to determine the sustained impacts. Including different age groups and both male and female athletes in future research will enhance the external validity and applicability of the findings. Additionally, long-term follow-up studies may help to more comprehensively assess the lasting effects of morning and evening exercise sessions.

## 5 Conclusion

In this study, participants were grouped based on their responses to the HS-MEQ scale, with the morning group training in the morning and the evening group training in the evening. The study involved male preadolescent football players aged 10–12 years. The findings indicate that, compared to evening runs, morning runs had a more significant impact on respiratory function, respiratory muscle strength, agility, and lower extremity strength in male football players within this age group, following an eight-week training program considering diurnal variation and chronotype. Based on these results, incorporating morning runs into the training regimen for male footballers aged 10–12 years is recommended to enhance their overall physical performance.

## Data Availability

The datasets presented in this study can be found in online repositories. The names of the repository/repositories and accession number(s) can be found in the article/supplementary material.
